# Supplementary material for: Characterizing the structural complexity of the Earth’s forests with spaceborne lidar
Source: Nat Commun. 2024 Sep 16;15:8116. doi: 10.1038/s41467-024-52468-2 (PMC11405527; doi:10.1038/s41467-024-52468-2)
Supplement: Supplementary file 1 — Supplementary Information [file 41467_2024_52468_MOESM1_ESM.pdf]

Characterizing the structural complexity of the Earth's forests with spaceborne lidar

Tiago de Conto, John Armston, Ralph Dubayah

Supplementary information

Supplementary Table 1. WSCI prediction intervals at 95% confidence level for each PFT model. Error = fraction of true targets not included in the prediction intervals; Efficiency = mean prediction interval size at 95% probability (two-sided), in WSCI units.

Plant Functional Type (PFT)	Error	Efficiency
Deciduous Broadleaf Trees (DBT)	0.0500	2.76
Evergreen Broadleaf Trees (EBT)	0.0496	1.82
Evergreen Needleleaf Trees (ENT)	0.0498	2.67
Grassland + Shrubland + Woodland (GSW)	0.0498	4.45

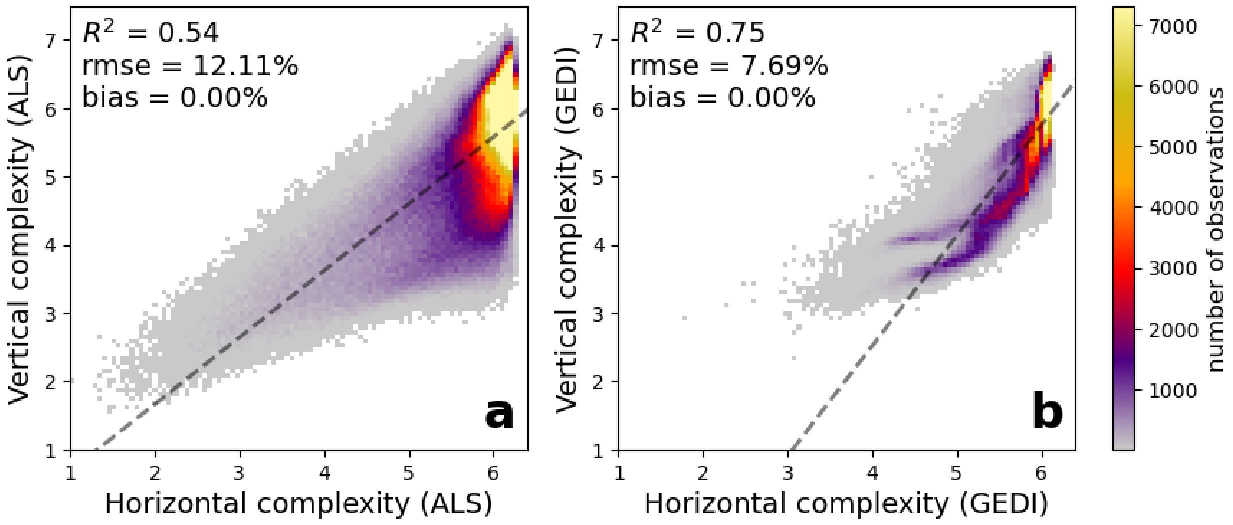

Supplementary Fig. 1. Relationship between horizontal and vertical complexity. Comparisons were made both in (a) observed airborne laser scanning (ALS) samples, and (b) model predictions from GEDI data.

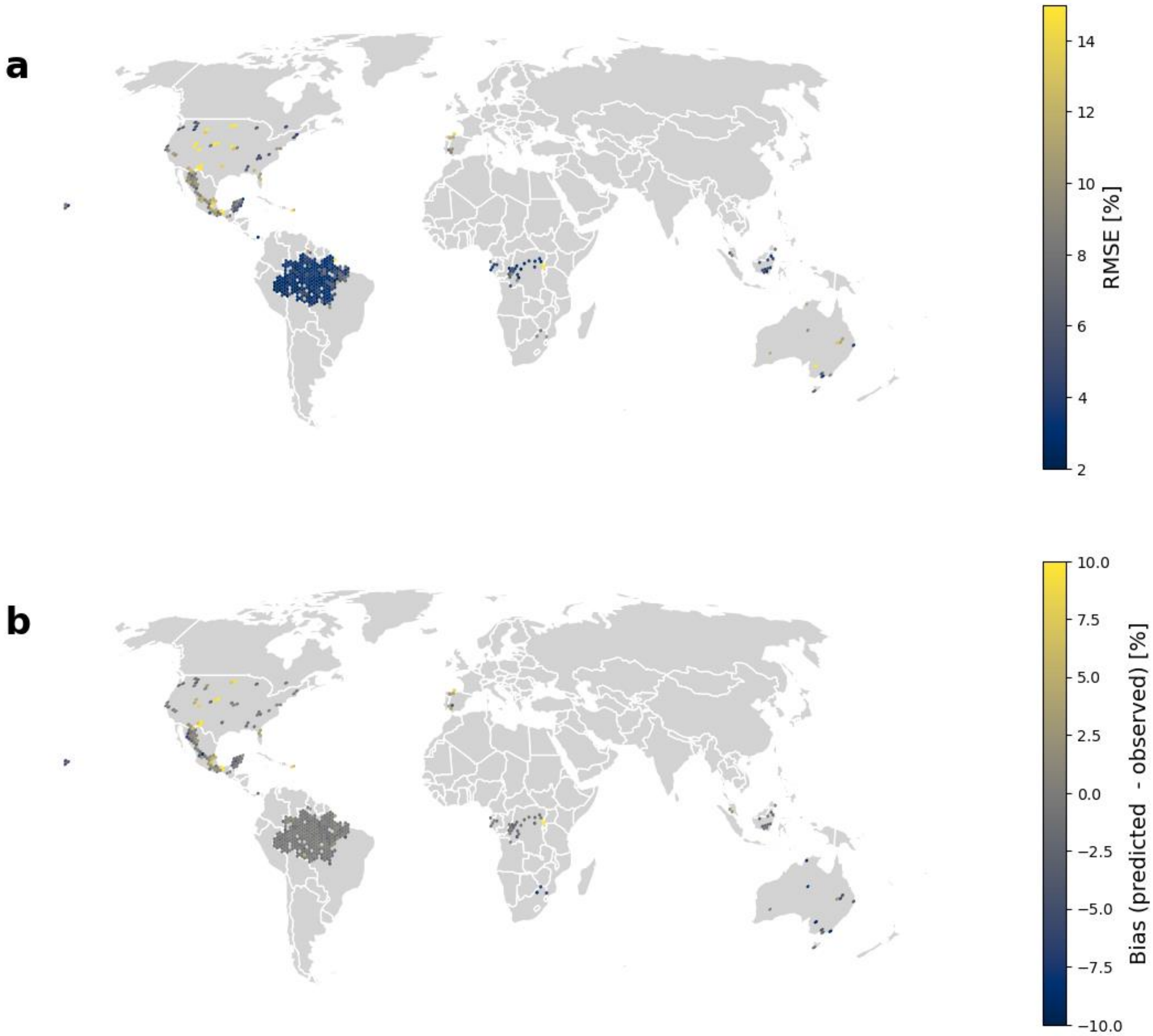

Supplementary Fig. 2. Geographical locations of 816,275 paired ALS/GEDI samples used for training the Waveform Structural Complexity Index (WSCI) models. (a) Relative root mean squared error and (b) absolute error (bias) were calculated from samples within hexagons of approximately 70 km radius, based on the predictions from the Plant Functional Type (PFT) model assigned to each sample.

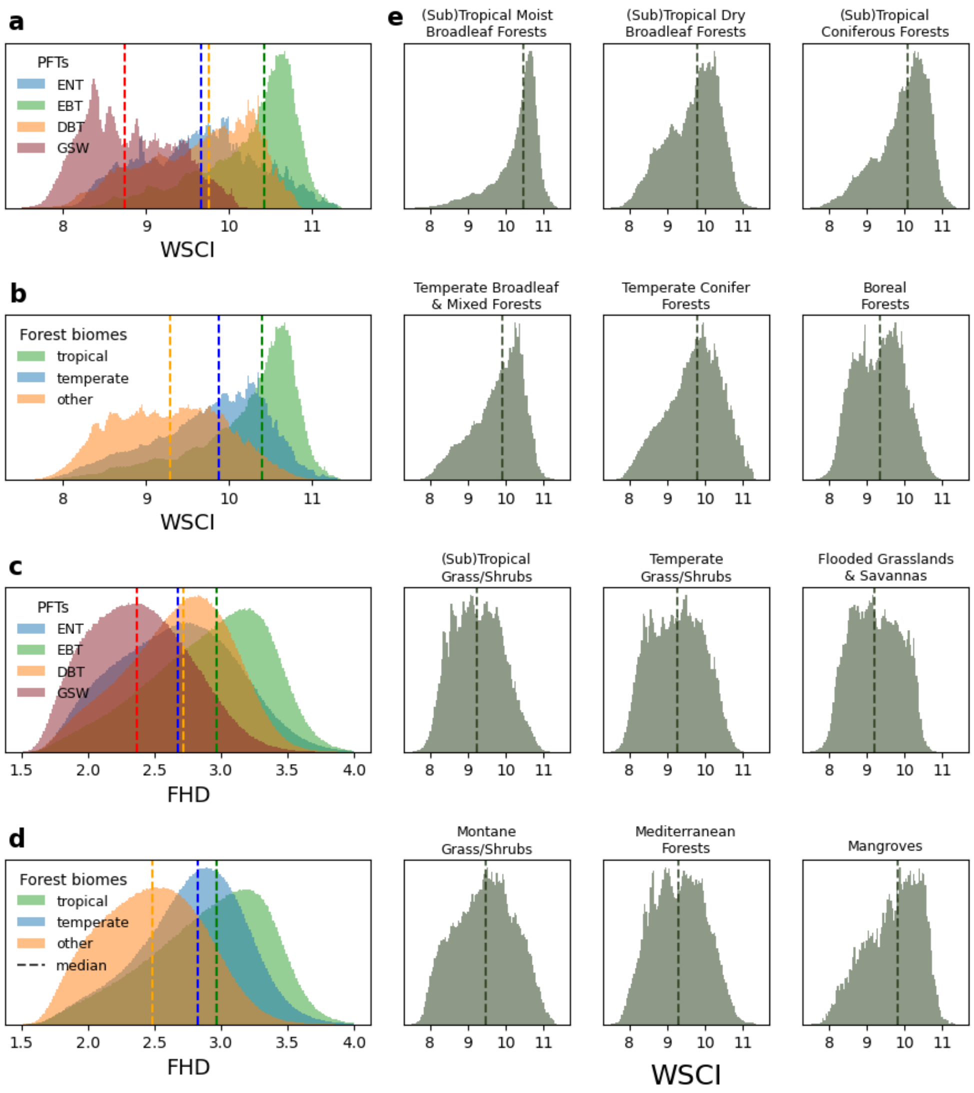

Supplementary Fig. 3. Global distributions of the Waveform Structural Complexity Index (WSCI) and Foliage Height Diversity (FHD) by Plant Functional Type (PFT) and biome. (a) Global frequency distributions of WSCI estimated by each PFT model. (b) Frequency distribution of WSCI over aggregated tropical, temperate and other forest biomes. (c) Frequency distributions of FHD by PFT. (d) . Frequency distribution of FHD over aggregated tropical, temperate and other forest biomes (e) Frequency distribution of WSCI within individual biomes. (ENT = Evergreen Needleleaf Trees, EBT = Evergreen Broadleaf Trees, DBT = Deciduous Broadleaf Trees, GSW = Grasslands, Shrublands and Woodlands).

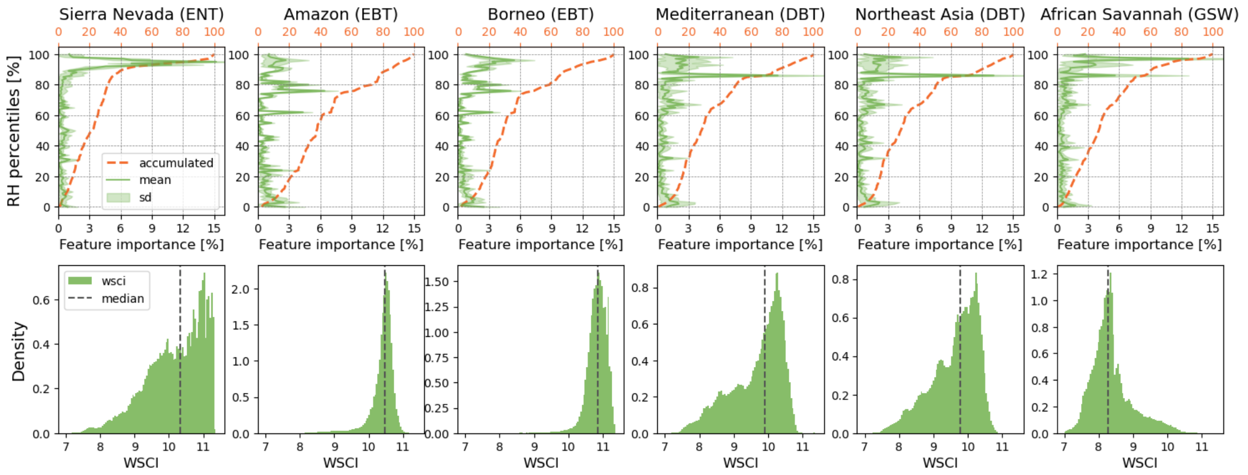

Supplementary Figure 4. Feature importance profiles and distribution of WSCI samples from forests belonging to different plant functional types (PFTs) and biomes. Temperate forests profiles were sampled from Sierra Nevada (United States), Mediterranean (southern Europe), and Northeast Asia; tropical forests were sampled in the Brazilian Amazon and Borneo (Southeast Asia); and samples from the African Savannah represent other biome types. (ENT = Evergreen Needleleaf trees, EBT = Evergreen Broadleaf trees, DBT = Deciduous Broadleaf Trees, GSW = Grasslands, Shrublands and Woodlands).

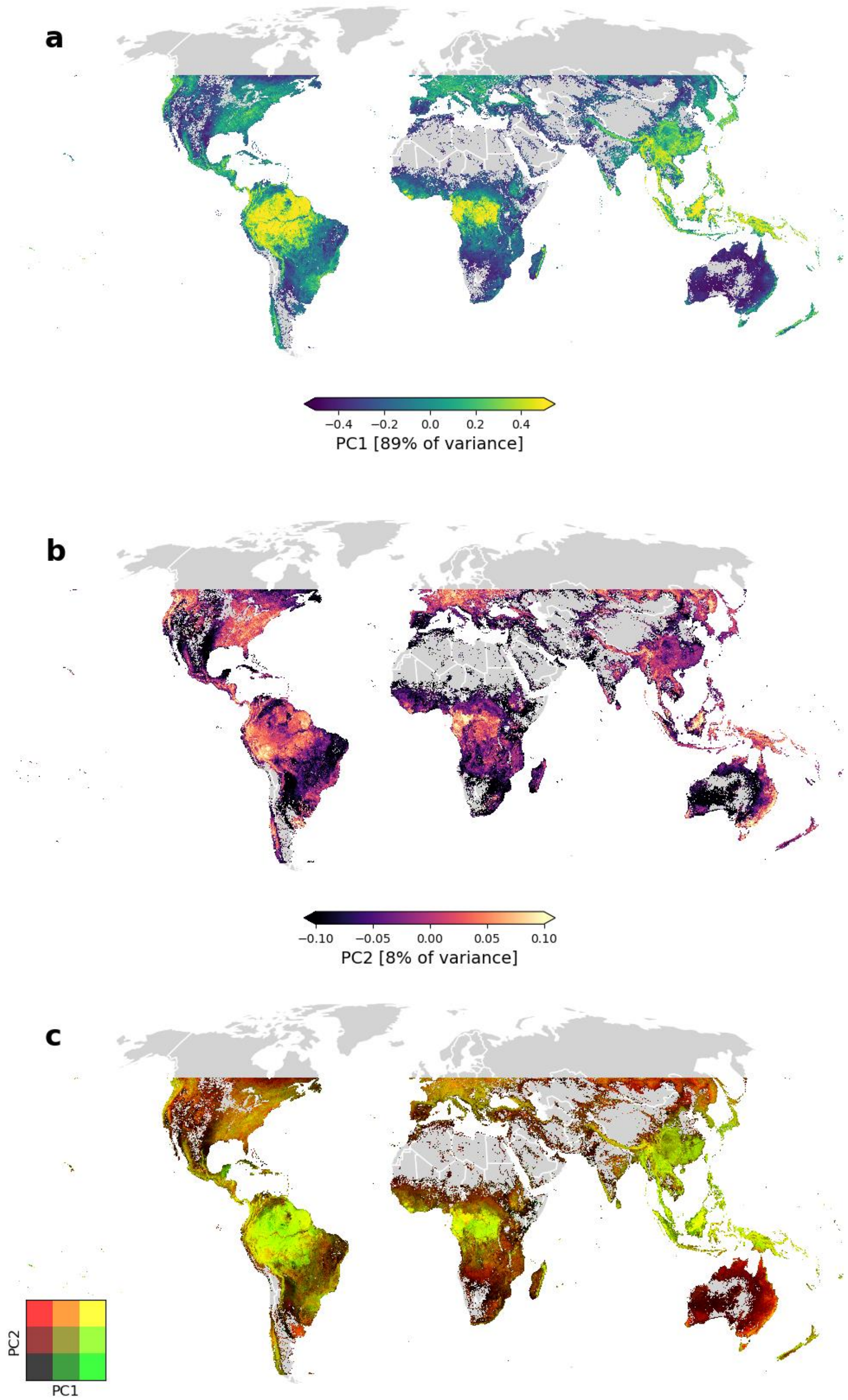

Supplementary Fig.5. Geographical patterns of Principal Components Analysis (PCA) scores averaged at 10 km resolution. (a) PC1 scores, (b) PC2 scores, (c) false color composite of PC1 (green) and PC2 (red).

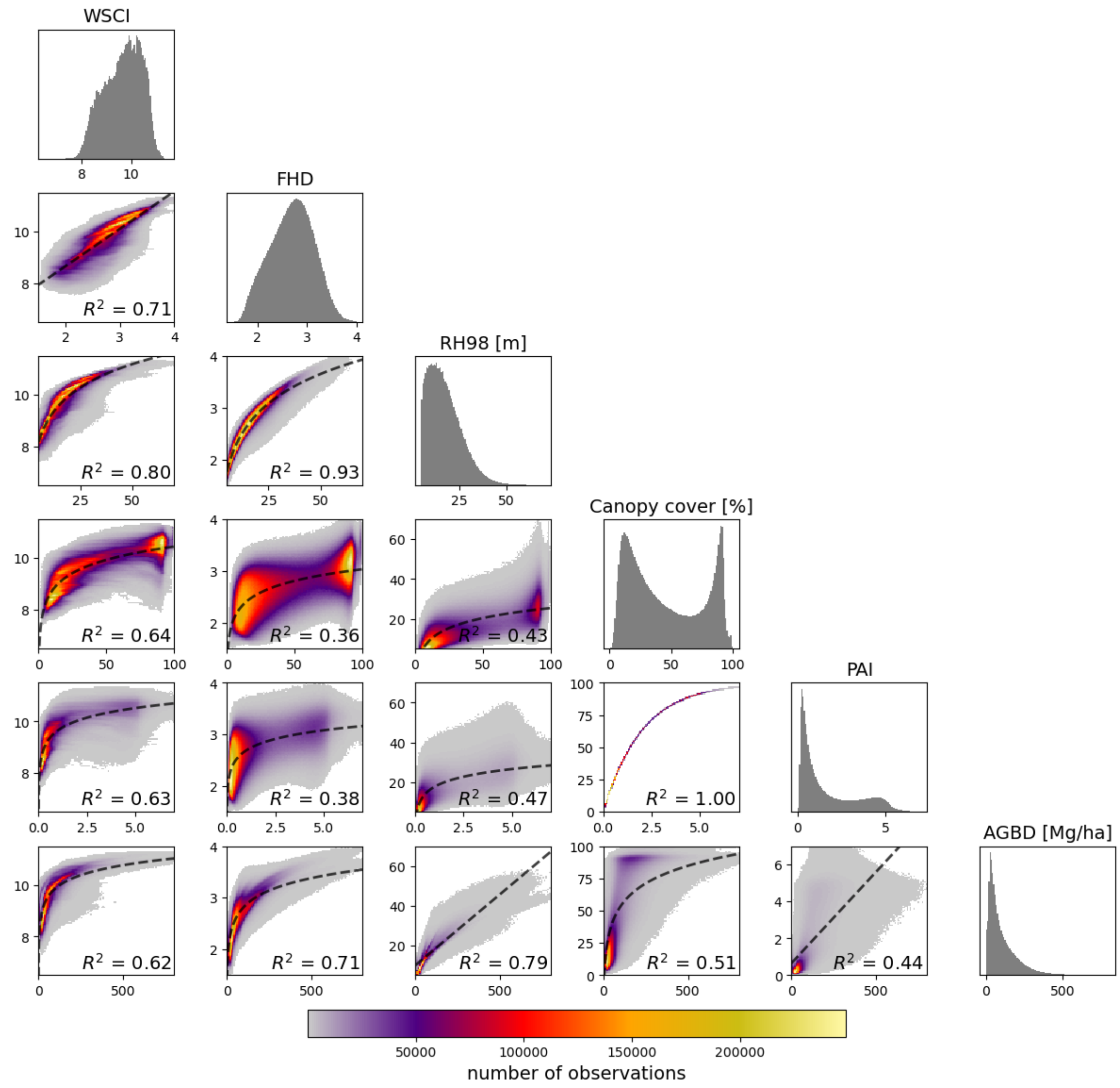

Supplementary Figure 6. Pairwise relationships of GEDI structural metrics. Note the R^2 greater than 0.6 for every pairwise comparison including the Waveform Structural Complexity Index (WSCI). FHD = Foliage Height Diversity, RH98 = canopy height, PAI = Plant Area Index, AGBD = Above Ground Biomass Density. PAI is a non-linear transformation of canopy cover, thus their pairwise R^2 = 1.

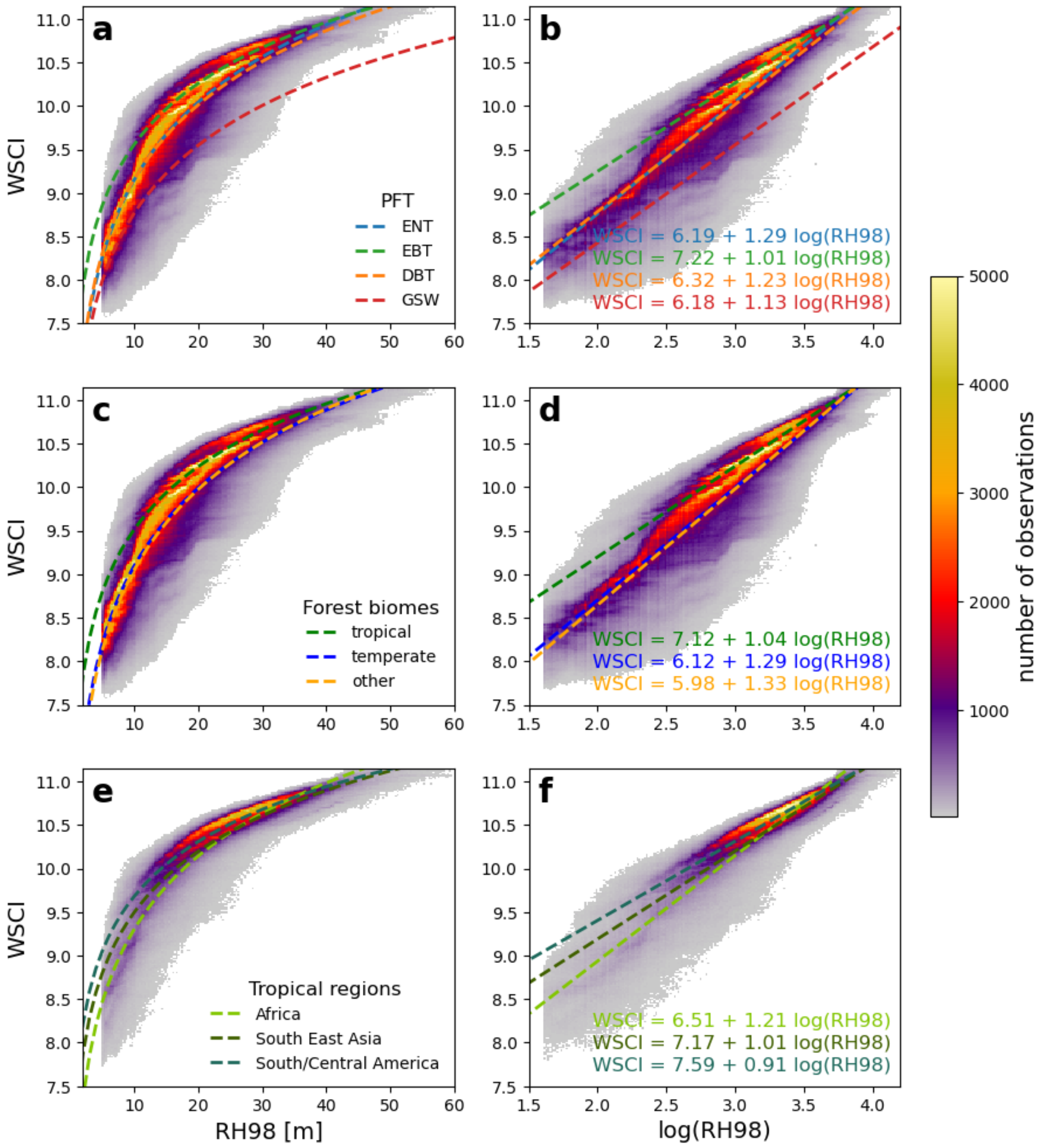

Supplementary Fig. 7. Scaling relationships between the Waveform Structural Complexity Index (WSCI) and canopy height (RH98). Complexity-height scaling was assessed across (a,b) plant functional types (PFTs), (c,d) aggregated biomes, and (e,f) between continental regions in the tropical biome. (ENT = Evergreen Needleleaf Trees, EBT = Evergreen Broadleaf Trees, DBT = Deciduous Broadleaf Trees, GSW = Grasslands, Shrublands and Woodlands).

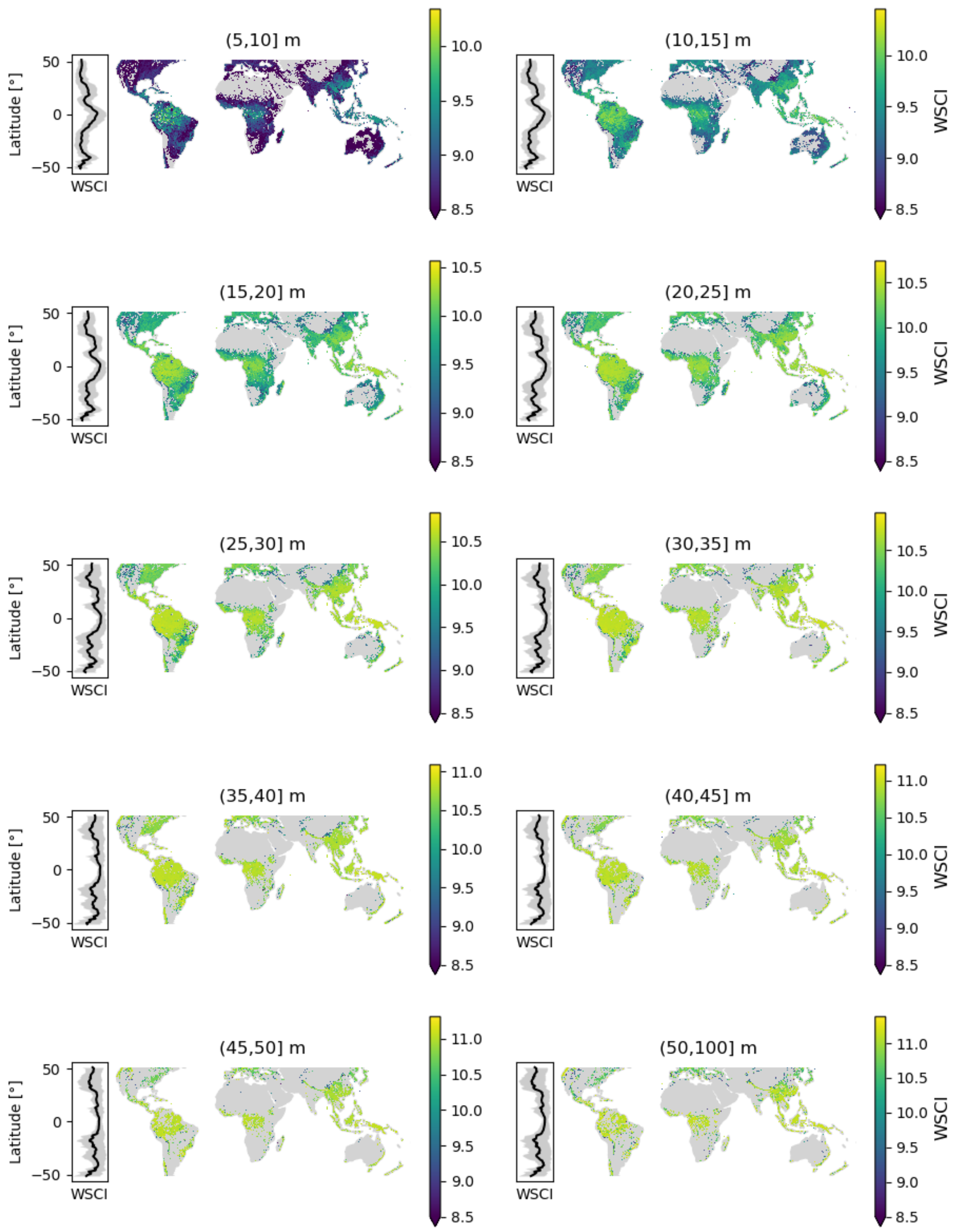

Supplementary Fig. 8. Geographical distributions of the Waveform Structural Complexity Index (WSCI) at different canopy heights. WSCI was averaged at 10 km resolution for all GEDI footprints where canopy top height (RH98) belonged to the height interval displayed in each subplot's title.

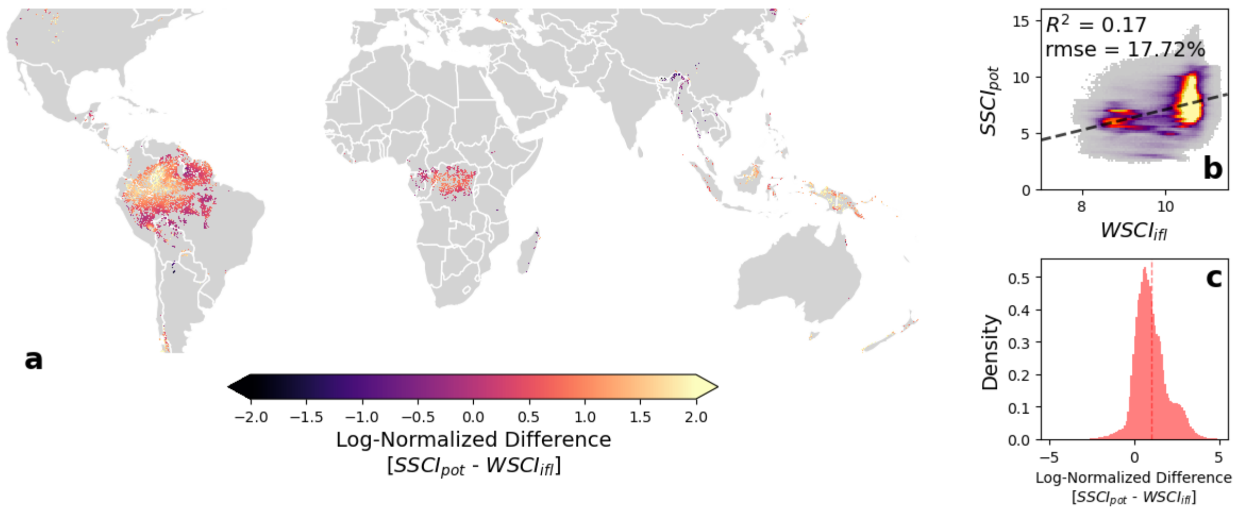

Supplementary Figure 9. Comparisons between potential stand structural complexity index ($SSCI_{pot}$) and average waveform structural complexity index at intact forest landscapes ($WSCI_{ifl}$) at 1 km scale. (a) Log-normalized mean difference of $SSCI_{pot}$ and $WSCI_{ifl}$, resampled from 1 km to 10 km pixels for better visualization, (b) linear relationship between $SSCI_{pot}$ and $WSCI_{ifl}$, and (c) distribution of the log-normalized difference of 1 km pixel values. Both metrics were log transformed and normalized to 0 mean and 1 standard deviation to be in the same range before comparisons.
