# Supplementary material for: Characterizing the structural complexity of the Earth’s forests with spaceborne lidar
Source: Nat Commun. 2024 Sep 16;15:8116. doi: 10.1038/s41467-024-52468-2 (PMC11405527; doi:10.1038/s41467-024-52468-2)
Supplement: Supplementary file 3 — Reporting Summary [file 41467_2024_52468_MOESM3_ESM.pdf]

nature portfolio

Corresponding author(s): Ralph Dubayah

Last updated by author(s): Aug 21, 2024

Reporting Summary

Nature Portfolio wishes to improve the reproducibility of the work that we publish. This form provides structure for consistency and transparency in reporting. For further information on Nature Portfolio policies, see our Editorial Policies and the Editorial Policy Checklist.

Statistics

For all statistical analyses, confirm that the following items are present in the figure legend, table legend, main text, or Methods section.

n/a	Confirmed	
☐	☒	The exact sample size (*n*) for each experimental group/condition, given as a discrete number and unit of measurement
☐	☒	A statement on whether measurements were taken from distinct samples or whether the same sample was measured repeatedly
☐	☒	The statistical test(s) used AND whether they are one- or two-sided<br>*Only common tests should be described solely by name; describe more complex techniques in the Methods section.*
☐	☒	A description of all covariates tested
☐	☒	A description of any assumptions or corrections, such as tests of normality and adjustment for multiple comparisons
☐	☒	A full description of the statistical parameters including central tendency (e.g. means) or other basic estimates (e.g. regression coefficient) AND variation (e.g. standard deviation) or associated estimates of uncertainty (e.g. confidence intervals)
☐	☒	For null hypothesis testing, the test statistic (e.g. *F*, *t*, *r*) with confidence intervals, effect sizes, degrees of freedom and *P* value noted<br>*Give P values as exact values whenever suitable.*
☒	☐	For Bayesian analysis, information on the choice of priors and Markov chain Monte Carlo settings
☒	☐	For hierarchical and complex designs, identification of the appropriate level for tests and full reporting of outcomes
☒	☐	Estimates of effect sizes (e.g. Cohen's *d*, Pearson's *r*), indicating how they were calculated

Our web collection on statistics for biologists contains articles on many of the points above.

Software and code

Policy information about availability of computer code

Data collection	no software was used
Data analysis	All data analysis was done using custom software. Point cloud processing code was implemented in the R language version 4.2.3, and CExyz calculation used packages lidR 4.1.0, TreeLS 2.0.5, ks 1.13.5, sf 1.0.12, nabor 0.5.0 and trend 1.1.4. WSCI models, conformal prediction and all statistical analyses were performed in the Python language, version 3.10.6, using packages xgboost 1.7.4, crepes 0.6.1, shap 0.43.0, statsmodels 0.14.0 and scikit-learn 1.3.2.

For manuscripts utilizing custom algorithms or software that are central to the research but not yet described in published literature, software must be made available to editors and reviewers. We strongly encourage code deposition in a community repository (e.g. GitHub). See the Nature Portfolio guidelines for submitting code & software for further information.

Data

Policy information about availability of data

All manuscripts must include a data availability statement. This statement should provide the following information, where applicable:

- Accession codes, unique identifiers, or web links for publicly available datasets
- A description of any restrictions on data availability
- For clinical datasets or third party data, please ensure that the statement adheres to our policy

GEDI data are openly available and archived on NASA Distributed Active Archive Centers (DAACs). The Waveform Structural Complexity Index (WSCI) data product is

openly available in the Oak Ridge National Laboratory (ORNL) DAAC as GEDI04_C Waveform Structural Complexity Index Product under accession code https://doi.org/10.3334/ORNLDAAC/2338. All Airborne Laser Scanning (ALS) datasets used in this study are available under open access and can be obtained directly from the sources or by contacting the principal investigators listed in Table 2 of this study. The GEDI footprint-level RH metrics used to train the WSCI models were taken from the GEDI02_A height and elevation product, available at the Land Processes (LP) DAAC under accession code https://doi.org/10.5067/GEDI/GEDI02_A.002. GEDI's FHD, PAI and cover metrics were taken from the GEDI02_B canopy cover and vertical profile metrics product also available at the LP DAAC under accession code https://doi.org/10.5067/GEDI/GEDI02_B.002. GEDI's footprint-level biomass data were taken from the GEDI04_A aboveground biomass density (AGBD) product, available at the ORNL DAAC under accession code https://doi.org/10.3334/ORNLDAAC/2056. The ESA worldcover v200 data product is available at https://worldcover2021.esa.int. The WWF Terrestrial Ecoregions of the World can be obtained at https://www.worldwildlife.org/publications/terrestrial-ecoregions-of-the-world.

Research involving human participants, their data, or biological material

Policy information about studies with human participants or human data. See also policy information about sex, gender (identity/presentation), and sexual orientation and race, ethnicity and racism.

Reporting on sex and gender	*Use the terms sex (biological attribute) and gender (shaped by social and cultural circumstances) carefully in order to avoid confusing both terms. Indicate if findings apply to only one sex or gender; describe whether sex and gender were considered in study design; whether sex and/or gender was determined based on self-reporting or assigned and methods used. Provide in the source data disaggregated sex and gender data, where this information has been collected, and if consent has been obtained for sharing of individual-level data; provide overall numbers in this Reporting Summary. Please state if this information has not been collected. Report sex- and gender-based analyses where performed, justify reasons for lack of sex- and gender-based analysis.*
Reporting on race, ethnicity, or other socially relevant groupings	*Please specify the socially constructed or socially relevant categorization variable(s) used in your manuscript and explain why they were used. Please note that such variables should not be used as proxies for other socially constructed/relevant variables (for example, race or ethnicity should not be used as a proxy for socioeconomic status). Provide clear definitions of the relevant terms used, how they were provided (by the participants/respondents, the researchers, or third parties), and the method(s) used to classify people into the different categories (e.g. self-report, census or administrative data, social media data, etc.) Please provide details about how you controlled for confounding variables in your analyses.*
Population characteristics	*Describe the covariate-relevant population characteristics of the human research participants (e.g. age, genotypic information, past and current diagnosis and treatment categories). If you filled out the behavioural & social sciences study design questions and have nothing to add here, write "See above."*
Recruitment	*Describe how participants were recruited. Outline any potential self-selection bias or other biases that may be present and how these are likely to impact results.*
Ethics oversight	*Identify the organization(s) that approved the study protocol.*

Note that full information on the approval of the study protocol must also be provided in the manuscript.

Field-specific reporting

Please select the one below that is the best fit for your research. If you are not sure, read the appropriate sections before making your selection.

☐ Life sciences ☐ Behavioural & social sciences ☒ Ecological, evolutionary & environmental sciences

For a reference copy of the document with all sections, see nature.com/documents/nr-reporting-summary-flat.pdf

Ecological, evolutionary & environmental sciences study design

All studies must disclose on these points even when the disclosure is negative.

Study description	We developed a waveform structural complexity index (WSCI) for GEDI waveforms through modeling a 3D complexity index from airborne laser scanning (ALS) point clouds collocated with GEDI footprints globally. We derived models for different plant functional types (PFTs). We then used WSCI estimates to characterize forest structural complexity patterns globally, how it relates to other structural attributes, what canopy elements describe complexity and how those vary geographically.
Research sample	All samples used in this study were recorded on forest lands. All ALS sites were recorded over forest sites, across 4 different PFT categories. All GEDI samples used in global assessments were further filtered to include tree cover only data. GEDI data is openly available at the NASA DAACs. ALS sites used in this study were all acquired after 2013 and are either open source or provided by authors of the studies for which the datasets were collected. All ALS sources are listed and referenced in Table 2.
Sampling strategy	Our training samples consist of GEDI footprints matched to ALS samples extracted from forest sites, producing a substantial training database of more than 800,000 samples evenly distributed across 4 PFT categories across the Earth. GEDI observations collected throughout the 4 years of the mission are randomly distributed in space. The entire GEDI data catalog between 2018 and 2023 was used in our study. We only excluded low quality and non-forest observations, using all the remaining data in our analyses.
Data collection	No data collection was done specifically for this study.

Timing and spatial scale	The scope of our study is the same as the GEDI mission, which continuously registered data between April 2018 and March 2023, between 51.6 degrees of latitude north and south.
Data exclusions	GEDI data was filtered to exclude low quality and non-forest observations. Bad quality data are usually related to atmospheric interferences or geolocation errors. Training data was further filtered to remove low fidelity matches between GEDI and ALS, further controlling for geolocation errors and substantial changes in the forest sites between ALS and GEDI acquisitions prior to model fitting. All filtering criteria and their rationale are fully described in the methods section of the paper.
Reproducibility	All data sources used to develop the WSCI are sourced and accessible by anyone. The WSCI product was also made available and is fully open source. Following the procedures described in this paper`s methods section and by having access to the same data and software resources makes the study fully reproducible.
Randomization	GEDI data has explicit geographical coordinates and PFT flags for each footprint, matched from the Modis Land Cover product. The different WSCI models were trained on a PFT basis using that information. Analyses at the biome level used the WWF ecoregions shapefile to assign the biome of each GEDI footprint used in this study. The GEDI orbital tracks intersections that happened overtime produced a large pool of observations randomly distributed over the Earth`s land mass. All high quality GEDI observations overlapping ALS sites were used in the study. Grid search hyperparameter tuning and spatial cross-validation were employed to enforce model generalization, controlling for factors such as collinearity of model inputs and geographical biases.
Blinding	Blinding is not relevant in our study. We used the entire GEDI catalog and the sample locations were the footprints geographical coordinates. We only filtered for quality observations and did not use any arbitrary selection criteria beyond that.

Did the study involve field work? ☐ Yes ☒ No

Reporting for specific materials, systems and methods

We require information from authors about some types of materials, experimental systems and methods used in many studies. Here, indicate whether each material, system or method listed is relevant to your study. If you are not sure if a list item applies to your research, read the appropriate section before selecting a response.

Materials & experimental systems

n/a	Involved in the study
☒	☐ Antibodies
☒	☐ Eukaryotic cell lines
☒	☐ Palaeontology and archaeology
☒	☐ Animals and other organisms
☒	☐ Clinical data
☒	☐ Dual use research of concern
☒	☐ Plants

Methods

n/a	Involved in the study
☒	☐ ChIP-seq
☒	☐ Flow cytometry
☒	☐ MRI-based neuroimaging

Plants

Seed stocks	*Report on the source of all seed stocks or other plant material used. If applicable, state the seed stock centre and catalogue number. If plant specimens were collected from the field, describe the collection location, date and sampling procedures.*
Novel plant genotypes	*Describe the methods by which all novel plant genotypes were produced. This includes those generated by transgenic approaches, gene editing, chemical/radiation-based mutagenesis and hybridization. For transgenic lines, describe the transformation method, the number of independent lines analyzed and the generation upon which experiments were performed. For gene-edited lines, describe the editor used, the endogenous sequence targeted for editing, the targeting guide RNA sequence (if applicable) and how the editor was applied.*
Authentication	*Describe any authentication procedures for each seed stock used or novel genotype generated. Describe any experiments used to assess the effect of a mutation and, where applicable, how potential secondary effects (e.g. second site T-DNA insertions, mosiacism, off-target gene editing) were examined.*
